# Synaptic Cell Adhesion Molecule 3 (SynCAM3) Deletion Promotes Recovery from Spinal Cord Injury by Limiting Glial Scar Formation

**DOI:** 10.3390/ijms23116218

**Published:** 2022-06-01

**Authors:** Byeong Gwan Song, Su Yeon Kwon, Jae Won Kyung, Eun Ji Roh, Hyemin Choi, Chang Su Lim, Seong Bae An, Seil Sohn, Inbo Han

**Affiliations:** 1 Department of Neurosurgery, CHA University School of Medicine, CHA Bundang Medical Center, Seongnam-si 13496, Gyeonggi-do, Korea; bgsong97@gmail.com (B.G.S.); syunkwon@naver.com (S.Y.K.); kyungjaewon88@gmail.com (J.W.K.); morolro@naver.com (E.J.R.); littlechoi88@gmail.com (H.C.); cslim8112@gmail.com (C.S.L.); anseongbae@gmail.com (S.B.A.); sisohn@cha.ac.kr (S.S.); 2 Department of Life Science, CHA University School of Medicine, Seongnam-si 13488, Gyeonggi-do, Korea

**Keywords:** spinal cord injury, cell adhesion molecules, synaptic adhesion molecules, astrocyte, glial scar, extracellular matrix

## Abstract

Synaptic cell adhesion molecules (SynCAMs) play an important role in the formation and maintenance of synapses and the regulation of synaptic plasticity. SynCAM3 is expressed in the synaptic cleft of the central nervous system (CNS) and is involved in the connection between axons and astrocytes. We hypothesized that SynCAM3 may be related to the astrocytic scar (glial scar, the most important factor of CNS injury treatment) through extracellular matrix (ECM) reconstitution. Thus, we investigated the influence of the selective removal of SynCAM3 on the outcomes of spinal cord injury (SCI). SynCAM3 knock-out (KO) mice were subjected to moderate compression injury of the lower thoracic spinal cord using wild-type (WT) (C57BL/6JJc1) mice as controls. Single-cell RNA sequencing analysis over time, quantitative real-time polymerase chain reaction (qRT-PCR) analysis, and immunohistochemistry (IHC) showed reduced scar formation in SynCAM3 KO mice compared to WT mice. SynCAM3 KO mice showed improved functional recovery from SCI by preventing the transformation of reactive astrocytes into scar-forming astrocytes, resulting in improved ECM reconstitution at four weeks after injury. Our findings suggest that SynCAM3 could be a novel therapeutic target for SCI.

## 1. Introduction

Axon regeneration and the sprouting processes that underlie plasticity are extremely limited in the injured adult spinal cord [1,2]. The main inhibitory molecules after spinal cord injury (SCI) are myelin-associated inhibitory molecules, including Nogo-A, myelin-associated glycoprotein, and oligodendrocyte myelin glycoprotein) and molecules found in association with glial scars [3,4,5,6,7,8]. Myelin-associated inhibitory molecules present in normal oligodendrocytes and myelin debris, resulting from the breakdown of myelin sheaths immediately after SCI, which are non-permissive substrates for neurite outgrowth [8,9]. The formation of chondroitin sulfate proteoglycans (CSPGs), semaphorin 3A, and collagen-based membranes become apparent after SCI and inhibit the regenerative processes of axons within the glial scar tissue of the lesion [9,10,11]. Therapeutic strategies to attenuate the activity of regeneration-inhibitory molecules and to induce axonal regeneration have led to some successful results.

For a neural circuit to be successfully remodeled, the growing collaterals not only need to reach their appropriate target cells, but also to form new synaptic connections. The formation of synapses requires the involvement of intersynaptic adhesion molecules that cover the synaptic cleft [12,13,14]. In vertebrates, this process is considered to be mediated via synaptic adhesion molecules. Synaptic cell adhesion molecules (SynCAMs), also known as nectin-like molecules (NECL) or cell adhesion molecules (CADMs), comprise a group of four immunoglobulin superfamily members that are crucial for the establishment of new synapses during development [15,16]. Interestingly, these molecules are also prominently expressed in the adult brain and spinal cord [17]. SynCAMs are transmembrane or membrane-tethered proteins involved in the maintenance, function, and elimination of synapses [16,18].

SynCAM3 is expressed in axons, where it interacts primarily with CADM4 and plays an important role in axon guidance, myelination, and the maintenance of the axonal architecture [19]. In the developing central nervous system (CNS), delayed myelination in the optic nerve and the spinal cord was found in SynCAM3 knock-out (KO) mice [16,20]. However, the removal of axonal SynCAM3 has been reported to promote Schwann cell myelination in the in vitro dorsal root ganglion neuron/Schwann cell myelinating system [21]. SynCAM3 can also be used for various CNS diseases as a biomarker. SynCAM3 has also been reported to inhibit glioma tumorigenesis by regulating histone deacetylation, to serve as a potential marker for the diagnosis of Alzheimer’s disease due to the significantly high expression of SynCAM3 in a pyroglutamate modified amyloid β-expressing transgenic mice model, and to be implicated in neuron degeneration caused by neuromuscular junction impairments and the reduction of extracellular matrix (ECM) integrity under conditions of low SynCAM3 expression [22,23,24].

In the synaptic cleft, SynCAM3 also facilitates the adhesion between the axon terminal and astrocytes [25,26]. Astrocytes in the tripartite synapse, which consist of the presynaptic neuron, postsynaptic neuron, and astrocytes, release neurotransmitters into the synaptic cleft and strengthen the signal of the synapse [27]. In addition to playing a beneficial role in the CNS, astrocytes carry out specific responses to damage. When SCI occurs, the naive astrocytes reactivated to become reactive astrocytes (RAs), which migrate to the lesion epicenter in a process known as astrogliosis to repair injured tissue in the acute phase [28,29]. However, RAs gradually transform into scar-forming astrocytes (SAs) in the chronic phase, leading to glial scar formation and the deposition of molecules that inhibit axon regeneration [6,30]. Type I collagen is one of the various factors contributing to the formation of glial scars. Its expression is increased in the chronic phase, transforming RAs into SAs via the integrin-N-cadherin pathway [30,31].

Based on these observations, SynCAM3 present at the site of SCI in the chronic phase has the potential to promote adhesion between axons and astrocytes and glial scar formation through the deposition of type I collagen. Therefore, we hypothesized that SynCAM3 may be involved in functional recovery after SCI and may be a novel therapeutic target for the treatment of SCI. To investigate the role of SynCAM3 in vivo, we conducted an animal experiment using SynCAM3 KO mice.

## 2. Results

### 2.1. SynCAM3, Whose Expression Levels Are Restored in the Chronic Phase, May Affect ECM Repositioning

In this study, we induced compression injuries (20 g for 1 min) at the 10th thoracic vertebrae level of wild-type (WT) mice and SynCAM3 knock-out (KO) mice using a metal impounder [32]. Although SynCAM3 has been reported in the spinal cord, brain, and dendritic spines, detailed localization studies have not been carried out. Therefore, we verified the presence of SynCAM3 in the injured spinal cord at 0, 2, 4, 7, 14, 21, and 28 days post-injury (DPI) [21,22,33].

**Figure 1:**
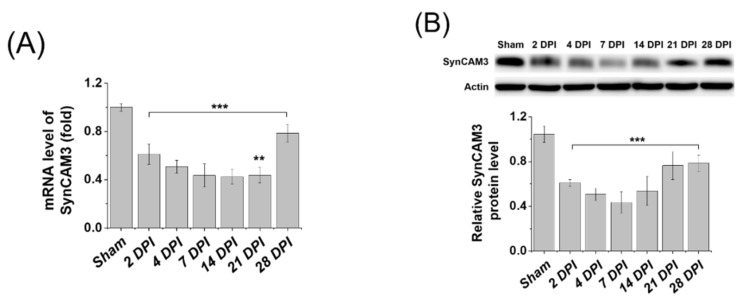

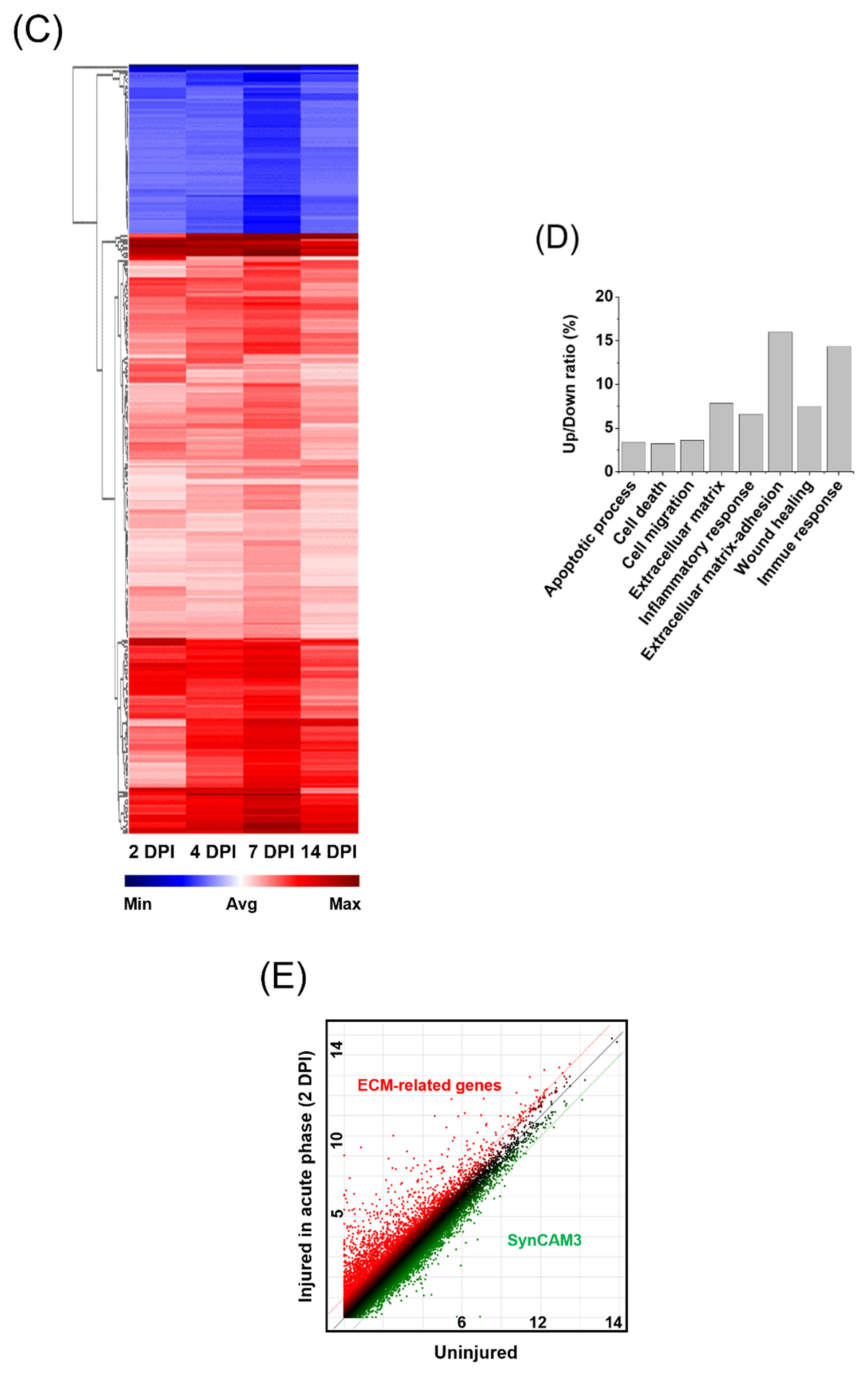
Identification of SynCAM3 and extracellular matrix (ECM)-related genes at multiple time points post compressive spinal cord injury (SCI). (**A**) Quantitative real-time polymerase chain reaction (qRT-PCR) showed decreased expression of SynCAM3 mRNA expression from day two to day 21 of spinal cord injury and restored expression on day 28 of injury. (*n* = 5 per time point). (**B**) Quantification of SynCAM3 protein expression revealed a decrease in expression up to seven days after injury and then started to recover (*n* = 5 per time point). (**C**) A heat map showing gene expression changes in the injured cord at 2, 4, 7, and 14 days post-injury (DPI). (**D**) Gene Ontology (GO) term analysis of overexpressed genes in the RNA-sequencing analysis of the injured cord (2 DPI) compared to those of the uninjured cord. The lists show the top eight upregulated/downregulated GO terms obtained, as ranked by the *p*-value. (**E**) Scatter plot of gene expression differences between the injured cords (2 DPI) and uninjured cords. ECM related genes were *Gfap*, *Bgn*, *Col1a1*, *Col1a2*, *Col3a1*, *Enl*, *F13a1*, *Ecm1*, *Tgfbi*, *and Mmp13*. Red or blue dots indicate genes that were significantly upregulated or downregulated, respectively. The data were presented as the mean ± SEM. ** *p* < 0.01 and *** *p* < 0.001 in one-way analysis of variance (ANOVA) with Dunnett’s test for multiple comparisons against the uninjured control group.

SynCAM3 expression significantly decreased in the acute phase (2–7 DPI), while it gradually recovered in the chronic phase (recovery phase), which was further confirmed by western blot and a quantitative real-time polymerase chain reaction (qRT-PCR) analysis (Figure 1A,B). We expected that SynCAM3, which recovered in the chronic phase after SCI, would become involved in the connection between axons and astrocytes, eventually leading to the formation of glial scars that interferes with spinal cord regeneration. Therefore, we hypothesized that the inhibition of SynCAM3 expression would suppress the formation of the glial scar in the chronic stage, which would aid in spinal cord regeneration. To demonstrate the effect of SynCAM3 deletion, we evaluated the relationship between SynCAM3 and gene expression in the injured cord. The heat map visually showed dynamic changes in the expression of all genes at 2, 4, 7, and 14 DPI (Figure 1C) [34,35]. It was confirmed that the most genetic changes occurred at seven DPI, when the expression of SynCAM3 was the lowest after SCI. This observation implies that gene expression in the injured cord seems to be associated with SynCAM3, which rapidly decreases after SCI. To find out which gene expression in the injured cord was associated with SynCAM3, Gene Ontology (GO) term analysis was performed at 2 DPI, when SynCAM3 expression decreased most rapidly. The GO term analysis indicated that the expression of genes involved in ECM adhesion, the immune response, and the ECM strongly upregulated (Figure 1D) [36]. The expression patterns of SynCAM3 and ECM-related genes were compared using a scatter plot, and it was found that the patterns were similar (Figure 1E). Collectively, these findings confirmed that the presence of SynCAM3 could affect ECM deposition, while SynCAM3 deletion could affect ECM repositioning.

### 2.2. Downregulation of Gene Expression Is Associated with ECM in the Lesion Area after SCI in SynCAM3 KO Mice

**Figure 2:**
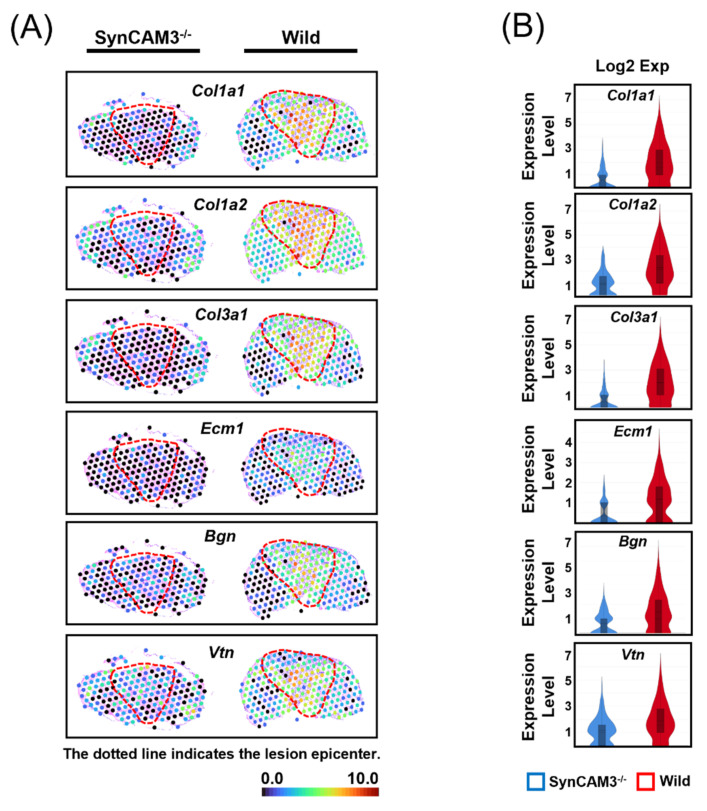

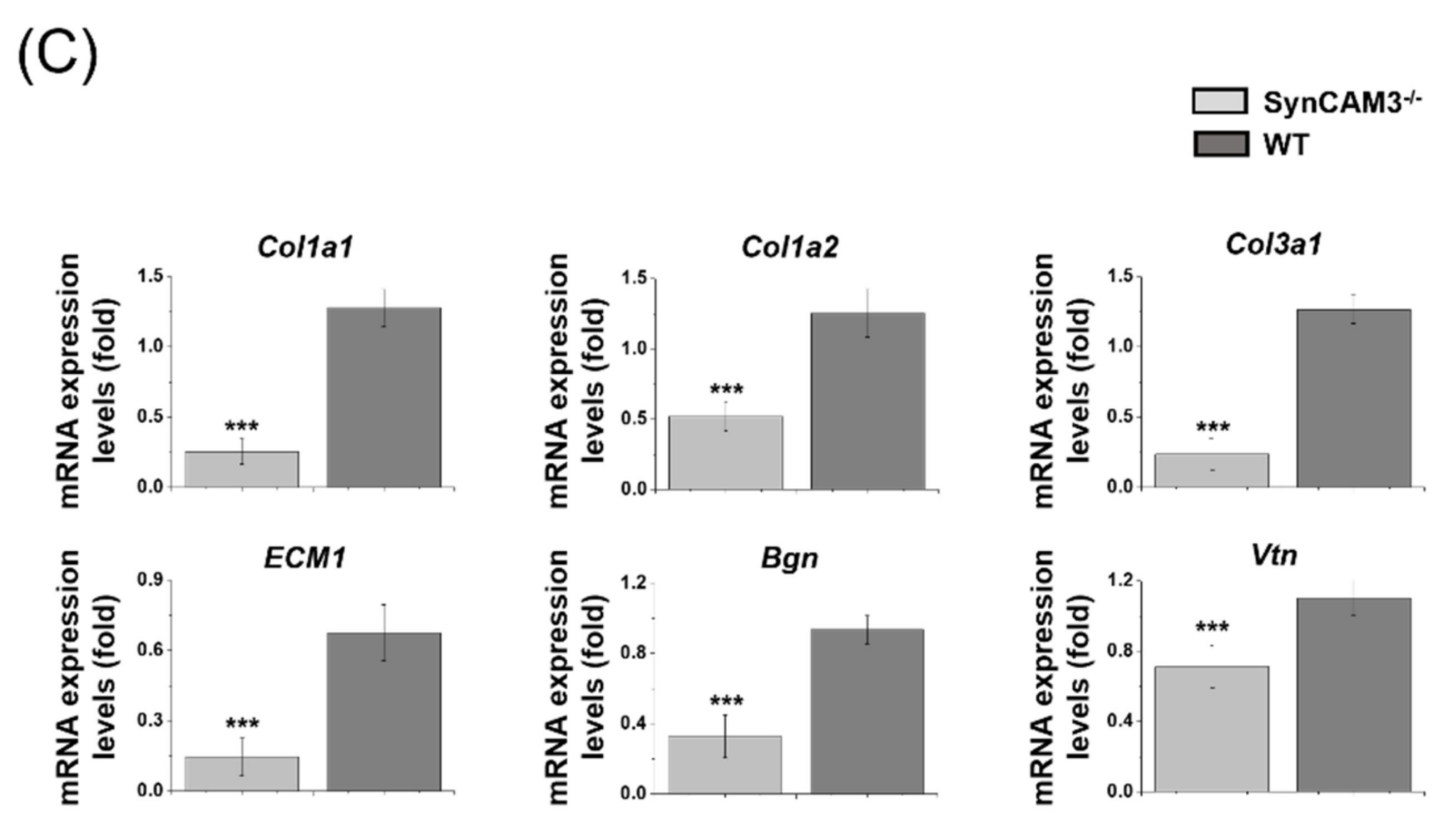
Spatiotemporal expression dynamics of *Col1a1*, *Col1a2*, *Col13a1*, *ECM1*, *Bgn*, and *Vtn*. (**A**) Spatial mRNA expression of *Col1a1*, *Col1a2*, *Col3a1*, *ECM1*, *Bgn*, and *Vtn* in WT mice and SynCAM3 KO mice at 28 DPI. (**B**) Violin plots of *Col1a1*, *Col1a2*, *Col13a1*, *ECM1*, *Bgn*, and *Vtn* expression in WT mice and SynCAM3 KO mice at 28 DPI, represented as log-normalized counts. (**C**) Quantification of mRNA expression levels for *Col1a1*, *Col1a2*, *Col13a1*, *ECM1*, *Bgn*, and *Vtn* in WT mice and SynCAM3 KO mice at 28 DPI. The data are presented as the mean ± SEM. *** *p* < 0.001 from one-way analysis of variance (ANOVA) with Dunnett’s test for multiple comparisons against the WT group.

To investigate the role of SynCAM3 in ECM matrix reposition in the chronic phase, we first performed spatial RNA sequencing (RNA-seq) at 28 DPI [37]. Our analysis revealed that ECM-related gene expression (*Col1a1*, *Col1a2*, *Col3a1*, *Ecm1*, *Bgn*, *and Vtn*) was lower in SynCAM3 KO mice than in WT mice (Figure 2). Those genes encode type I collagen and ECM molecules that could trigger astrocytic scars or modulate ECM reconstitution [38,39,40]. These findings indicate that SynCAM3 deletion could play an important role in ECM reconstitution in the chronic phase. In particular, these observations suggest that selective ablation of SynCAM3 could reduce the expression of collagen I-related gene (*Col1a1*, *Col1a2*) expression and inhibit glial scar formation in the chronic phase.

### 2.3. SynCAM3 Deletion Inhibits the Transformation of RAs into SAs through ECM-Adhesion Regulation in SCI

**Figure 3:**
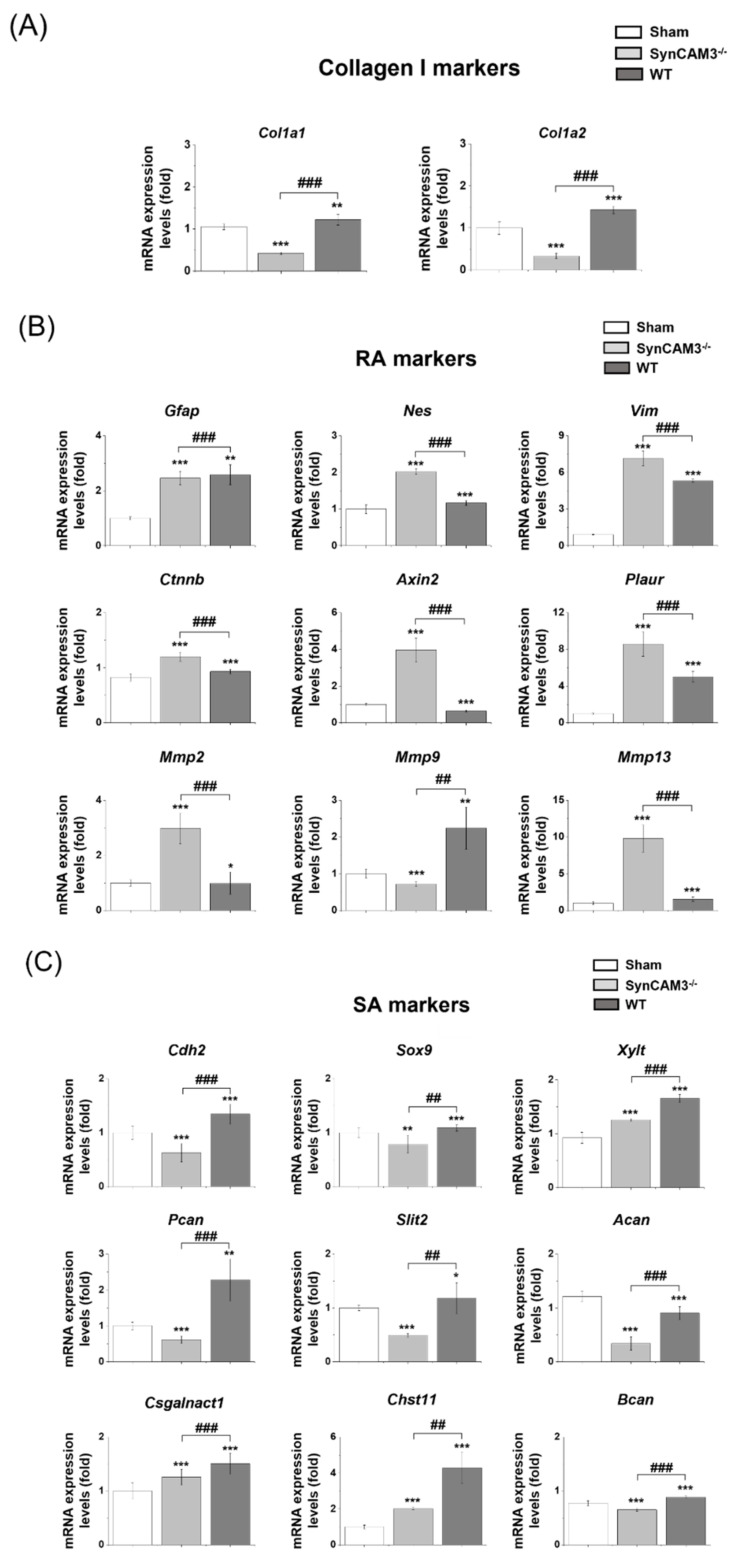
mRNA expression for markers of type I collagen, reactive astrocytes (RAs), and scar-forming astrocytes (SAs). (**A**) Quantification of mRNA expression levels for *Col1a1* and *Col1a2* in wild type (WT) mice and SynCAM3 knock-out (KO) mice at 28 DPI (*n* = four mice per group). (**B**) Quantification of mRNA expression levels for RA-related genes in WT mice and SynCAM3 KO mice at 28 DPI (*n* = four mice per group). (**C**) Quantification of mRNA expression levels for SA-related genes. * *p* < 0.05, ** *p* < 0.01 and *** *p* < 0.001 against the sham group. ## *p* < 0.01 and ### *p* < 0.001 (SynCAM3 KO vs. WT). The statistical analysis was performed using ANOVA with the Tukey–Kramer *post hoc* test.

Type I collagen was highly expressed in the injured spinal cord and induced astrocytic scar formation during the chronic phase. Therefore, we quantified the mRNA expression level of type I collagen-related genes, RA marker genes (*Gfap*, *Nes*, *Vim*, *Ctnnb*, *Axin2*, *Plaur*, *Mmp2*, *Mmp9*, and *Mmp13*), and SA marker genes (*Cdh2*, *Sox9*, *Xylt*, *Pcan*, *Slit2*, *Can*, *Csgalnact1*, *Chat11*, and *Bcan*) to assess the effect of SynCAM3 deletion on scar formation by qRT-PCR at 28 DPI. We observed the decreased expression of type I collagen -associated genes (e.g., *col1a1*, *col1a2*) in SynCAM3 KO mice compared to WT mice and sham mice, confirming that the deletion of SynCAM3 attenuated the expression of type I collagen (Figure 3A). Therefore, we expected that glial scar formation would be inhibited by the deletion of SynCAM3. The expression of RA marker genes (*Nes*, *Vim*, *Ctnnb*, *Axin2*, *Plaur*, *Mmp2*, *and Mmp13*) indicating tissue repair and functional improvement was higher in SynCAM3 KO mice than in WT mice at 28 DPI (Figure 3B). Additionally, the expression of SA marker genes (*Cdh2*, *Sox9*, *Xylt*, *Pcan*, *Slit2*, *Can*, *Csgalnact1*, *Chat11*, *and Bcan*) associated with glial scar formation was lower in SynCAM3 KO mice than in WT mice at 28 DPI, suggesting that SynCAM3 KO mice showed reduced glial scarring and better neurological improvement (Figure 3C). However, the expression of *Gfap* and *Mmp9* of RA markers was not significantly different between SynCAM3 KO mice and WT mice. It can be explained that these genes usually show high expression levels in both RAs and SAs. Therefore, it was assumed that WT mice, in which high expression of SA markers would be expected, would have higher expression than SynCAM3 KO mice, in which high expression of RA markers would be expected [41,42]. These findings indicate that SynCAM3 deletion inhibits the transformation of RAs into SAs through ECM-adhesion regulation in SCI.

### 2.4. SynCAM3 Deletion Inhibits Glial Scar Formation

**Figure 4:**
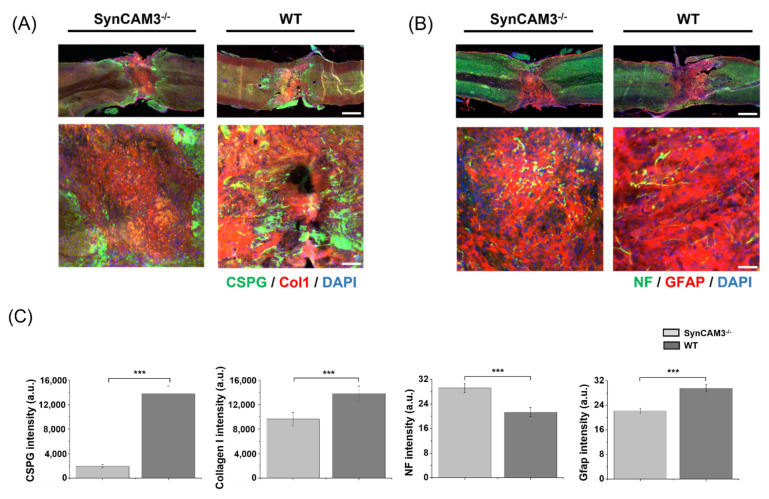
Reduced glial scar formation in SynCAM3 KO mice. (**A**) Representative images of immunofluorescence staining for glial scar-related components (chondroitin sulfate proteoglycans, green) and ECM-related components (Col1, red) in WT mice and SynCAM3 KO mice at 28 DPI (*n* = five animals per group). Scale bars, 500 μm (above), and 100 μm (below). (**B**) Representative images of immunofluorescence staining for neurons (neurofilaments, NF, green) and astrogliosis glial fibrillary acidic protein (GFAP, red) in WT mice and SynCAM3 KO mice at 28 DPI (*n* = five animals per group). Scale bars, 500 μm (above), and 50 μm (below). (**C**) Bar charts represent the intensity mean value (fluorescence) for the corresponding protein in the randomly selected field area at the injury epicenter in WT mice and SynCAM3 KO mice at 28 DPI (3 fields/slide, *n* = 3/group). Data are mean ± SEM. *** *p* < 0.001 (SynCAM3 KO vs. WT).

To assess the effects of SynCAM3 deletion on glial scarring, we performed immunofluorescence analysis of the injured spinal cords of the mice at 28 DPI. A glial scar contains ECM that is deposited by reactive astrocytes in response to SCI. The main components of the ECM deposited in glial scars are CSPGs, macromolecules that block other substances from the outside of the lesion [28,30,40,43]. The lesions of WT mice contained large amounts of CSPGs and type I collagen, hallmarks of the fibrotic scars that hinder axon regeneration, gradually increasing toward the lesion epicenter compared to SynCAM3 KO mice, and SynCAM3 KO mice showed lower deposition of CSPGs and type I collagen (Figure 4). In addition, the expression of glial fibrillary acidic protein (GFAP), indicative of glial scar formation, was lower in SynCAM3 KO mice, and the expression of neurofilament (NFs) of the indicative neuronal cytoskeleton, increasing toward the lesion epicenter, was higher in SynCAM3 KO mice than in WT mice at 28 DPI. These results showed that SynCAM3 KO mice had attenuated glial scarring.

### 2.5. Reduction of Microglial Activation in SynCAM3 KO Mice

**Figure 5:**
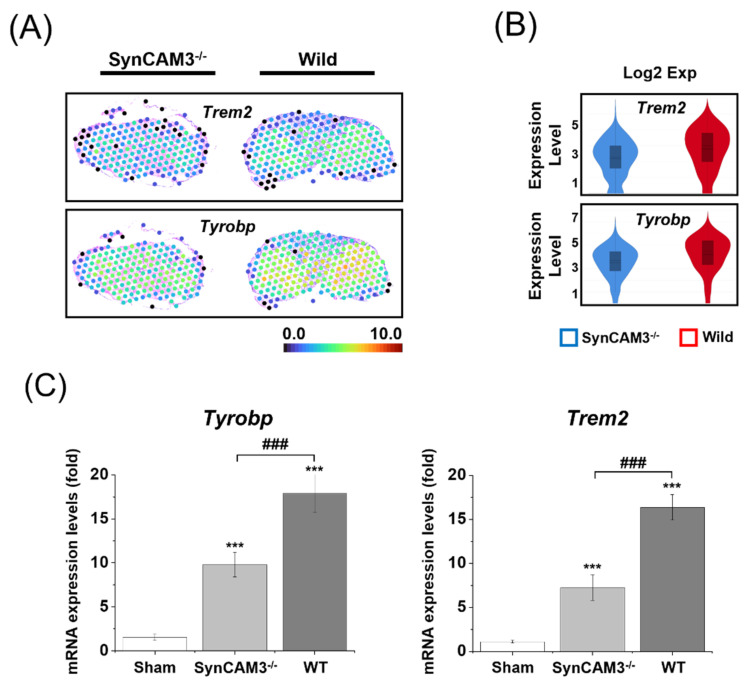
Spatiotemporal expression dynamics of *Trem2* and *Tyrobp*. (**A**) Spatial information on the mRNA expression of *Trem2* and *Tyrobp* in WT mice and SynCAM3 KO mice at 28 DPI. (**B**) Temporal downregulation of *Trem2* and *Tyrobp* in WT mice and SynCAM3 KO mice at 28 DPI is visualized as a violin plot. (**C**) Quantification of mRNA expression levels for the microglial activation-related genes *Trem2* and *Tyrobp* in WT mice and SynCAM3 mice at 28 DPI. The data are presented as the means ± SEM. *** *p* < 0.001 in one-way ANOVA with Dunnett’s test for multiple comparisons against the sham group. ### *p* < 0.001 (SynCAM3 KO vs. WT).

To further explore the spatiotemporal dynamics of microglial activation, we focused on the mechanism involving triggering receptors expressed on myeloid cells 2 (TREM2), which has been reported in neurodegenerative disease models. TREM2 and the transmembrane immune signaling adaptor (TYRO protein tyrosine kinase-binding protein, TYROBP) complex modulate pro-inflammatory cytokines (e.g., tumor necrosis factor [TNF]-α, interleukin [IL]-1β, and IL-6) and trigger phagocytosis. Microglial stimuli of apoptotic neurons activate this mechanism [44,45]. Our spatial gene expression analysis showed that the expression of *Trem2* and *Tyrobp*, inducing microglial activation, was upregulated specifically in WT mice, but not in SynCAM3 KO mice (Figure 5). In addition, the mRNA expression levels of *Trem2* and *Tyrobp* decreased in SynCAM3 KO mice (Figure 5B). Taken together, these results suggest that SynCAM3 deletion reduces the activity of microglia activation and the possibility of causing more severe spinal cord damage.

### 2.6. Appropriate Regulation of SNAP-25 Expression Induces Sensory and Locomotor Function in SCI

**Figure 6:**
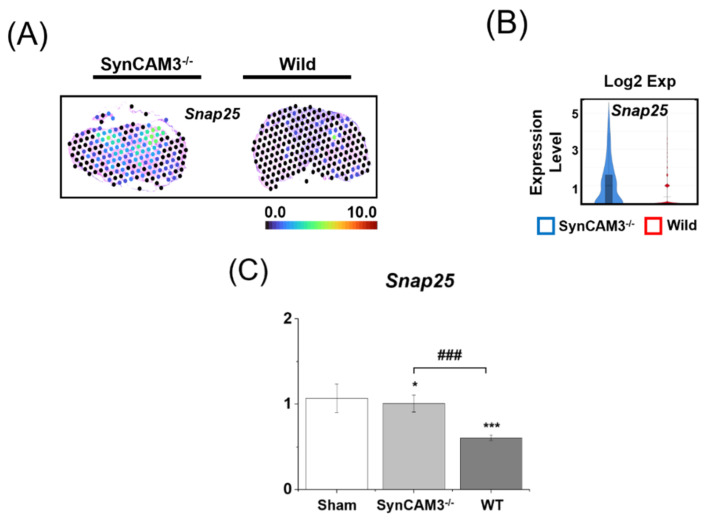
Spatiotemporal expression dynamics of *Snap25*. (**A**) Spatial mRNA patterns of *Snap25* expression in the spinal cord of WT mice and SynCAM3 KO mice at 28 DPI. (**B**) Temporal upregulation of *Snap25* in WT mice and SynCAM3 KO mice at 28 DPI is visualized as a violin plot. (**C**) Quantification of mRNA expression levels for the synaptic plasticity-related gene *Snap25* in WT mice and SynCAM3 KO mice at 28 DPI. The data are presented as the means ± SEM. * *p* < 0.05, and *** *p* < 0.001 in one-way ANOVA with Dunnett’s test for multiple comparisons against the sham group. ### *p* < 0.001 (SynCAM3 KO vs. WT).

Synaptosomal-associated protein-25 (SNAP-25) is a component of the SNARE (SNAP receptor) complex, which is closely related to repair and regeneration after SCI. Previous studies have suggested that the low expression of SNAP-25 affects recovery and motor function [46,47]. However, other studies have reported that the high expression of SNAP-25 promoted neurite outgrowth and regulated sensory function recovery, and that low expression of SNAP-25 caused sensory deficits in the spinal cord [48,49]. Since the mechanism of SNAP-25 remains unclear, we assumed that expression at a similar level to the sham group would be effective for spinal cord regeneration. Therefore, we carried out spatial and temporal mRNA expression analyses to confirm the effect of SynCAM3 deletion on SNAP-25. We identified an increase in the expression of *Snap25* in SynCAM3 KO mice compared to WT mice (Figure 6). In addition, the mRNA expression level of *Snap25* in SynCAM3 KO mice was similar to that in the sham group. Accordingly, the deletion of SynCAM3 may be essential for regulating regeneration after SCI.

### 2.7. SynCAM3 Deletion Quickly Restores Myelination and Dysfunction of the Spinal Cord

**Figure 7:**
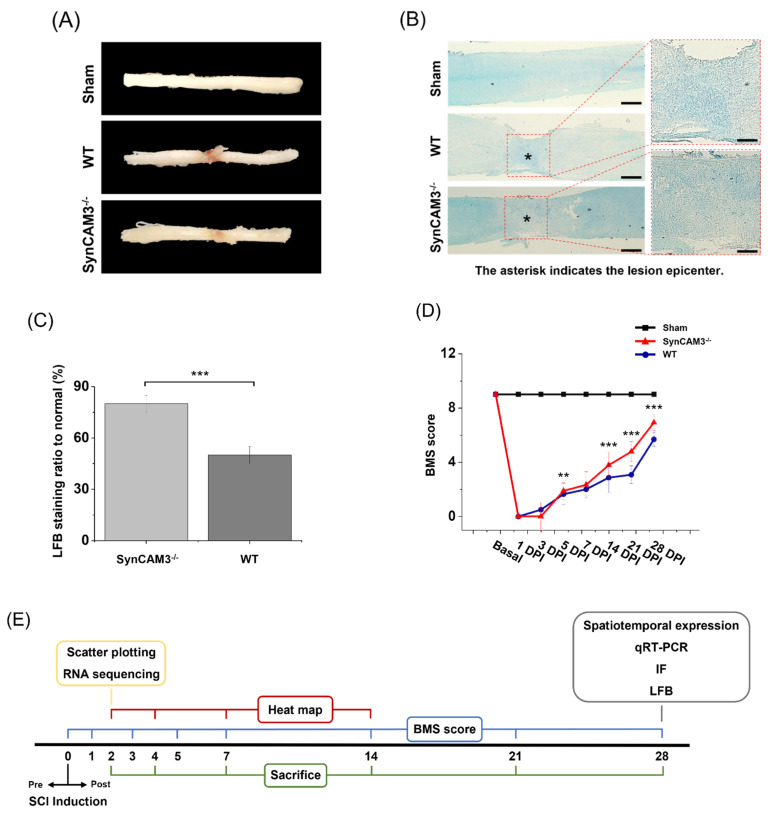

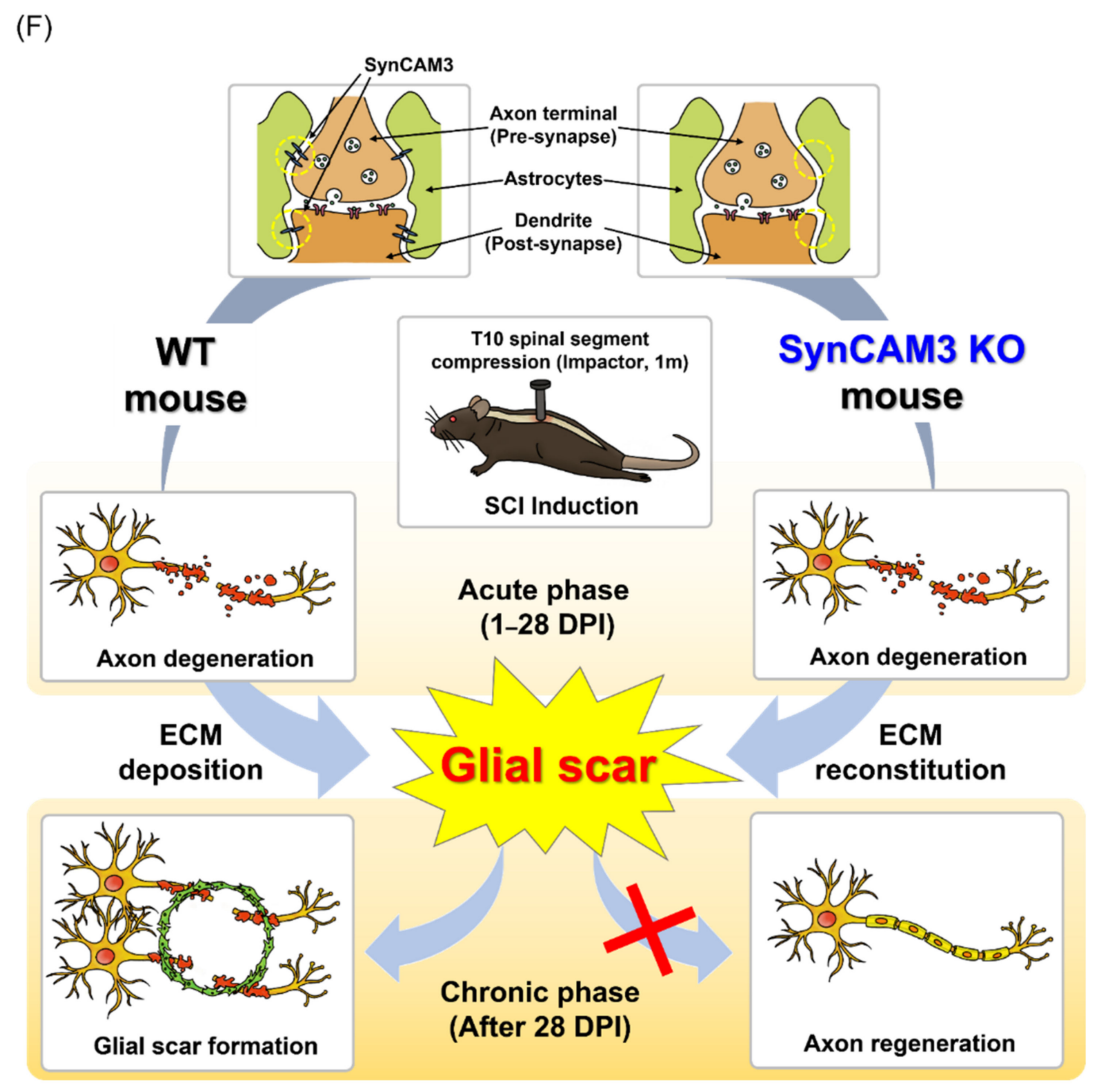
Improved functional recovery and remyelination in SynCAM3 mice. (**A**) Gross morphology of the spinal cord at 28 DPI. (**B**) Luxol fast blue (LFB) staining in WT mice and SynCAM3 KO mice at 28 DPI. (**C**) Quantification result of LFB staining in WT mice and SynCAM3 KO mice at 28 DPI. Scale bars were 500 μm and 100 μm on left and right (expanded image). (**D**) BMS score-based quantitative analysis of time-lapse functional recovery in spinal cord-injured mice and representative images of injured animals at 28 DPI. (**E**) Schematic of the timeline of the entire experiment. (**F**) Schematic of the experimental design for SCI. The data are presented as means ± SEM. ** *p* < 0.01 and *** *p* < 0.001 (SynCAM3 KO vs. WT). Two-way ANOVA was followed by the post hoc Bonferroni test.

To evaluate remyelination, Luxol fast blue staining (LFB) was performed. SynCAM3 KO mice showed more remyelination compared to WT mice (Figure 7A–E). It was confirmed that the external spinal cord was restored. To evaluate functional recovery under conditions of SynCAM3 deletion, we assessed motor function in SynCAM3 KO mice and WT mice using the Basso mouse scale (BMS) score at 0–28 DPI. The BMS score is commonly used to assess functional recovery following SCI in mice. Before SCI, the BMS scores for all mice were high. After the first postoperative day, the scores were low and the mice exhibited paraplegia, indicating successful modeling of SCI. The SynCAM3 KO mice scores at each time point were significantly higher than those of the WT mice (*n* = 8 mice/group) (Figure 7D). The hind limbs of the SynCAM3 KO mice indicated considerable walking ability, whereas the WT mice exhibited no ankle movement. Subsequently, the locomotor functional recovery in the SynCAM3 KO mice was significantly improved. These results demonstrated that functional recovery after static compression SCI was improved by the deletion of SynCAM3.

## 3. Discussion

In this study, we investigated the effects of SynCAM3 deletion targeting glial scar formation after SCI. SynCAM3 deletion reduced the expression of SAs, inhibited deposition of CSPG, type I collagen, and GFAP, which cause glial scar formation in the chronic phase, and restored motor functions after SCI through single-cell RNA-seq analysis over time, qRT-PCR analysis, immunofluorescence analysis, and behavior testing.

When a CNS injury occurs, RAs gradually transform into SAs and induce the formation of a glial scar, which forms a barrier between the normal and lesion area in the chronic phase. Astrocytic scars are widely regarded as a principal cause of axonal regrowth failure and poor functional outcomes [7]. Several attempts to treat SCIs in experimental rodent models focused on either improving axonal growth through the lesion site, modulating the glial scar tissue to a more growth-permissive state, or targeting combinations of inhibitory molecules (Nogo-A, myelin-associated glycoprotein, oligodendrocyte myelin glycoprotein), but various therapeutic challenges in attempts to decrease the activities of inhibitory molecules and promote neuronal regeneration have failed [4,5,6,50].

To inhibit glial scar formation by decreasing the activity of inhibitory molecules, we focused on SynCAM3, also known as NECL or CADM3, which interacts with axons and astrocytes in the synaptic cleft [25,26]. SynCAM3 expression decreased rapidly in the acute phase and was restored in the chronic phase, and an experiment was conducted to investigate the effect of SynCAM3 deletion on inhibition of glial scars and the regeneration of the spinal cord. Through GO term analysis, it was confirmed that SynCAM3 and spinal cord-related genes were related. In particular, ECM-related genes showed a similar expression pattern to SynCAM3. Additionally, we assessed the expression of ECM-related genes through spatial mRNA analysis at 28 DPI and confirmed the possibility that SynCAM3 deletion may play a role in ECM reconstitution with less expression compared to WT mice. Among ECM-related genes, the expression of type I collagen-related genes (*Col1a1*, *Col1a2*), triggering glial scars, was lower in the SynCAM3 KO mice than in the WT mice [30,31,38,39,40]. Based on these results, we expected that the removal of SynCAM3, which forms connections between axons and astrocytes, would inhibit glial scar formation through ECM reconstitution in the recovery phase. Additionally, the overexpression of RA markers and the down-expression of SA markers in SynCAM3 KO mice were identified through qRT-PCR. Immunofluorescence analysis was performed to identify glial scars; specifically, the expression of CSPG, collagen I, and GFAP was significantly lower, and the expression of NFs related to nerve regeneration was higher in SynCAM3 KO mice [40,43]. From the above results, we confirmed that SynCAM3 deletion can inhibit the formation of glial scars in the chronic phase. Our analysis of immune response-related genes also confirmed that the expression of *Trem2* and *Tyrobp*, which secrete inflammatory cytokines (e.g., TNF- α, IL-1β, IL-6) and lead to the death of neurons, was low; furthermore, spatial mRNA analysis showed that the expression of Snap25, which is related in sensory function recovery, was proper (i.e., similar to the control group) in SynCAM3 KO mice [45,46,47,48,49]. Lastly, we confirmed that SynCAM3 KO mice recovered motor function faster than WT mice through the BMS score.

In summary, our study establishes for the first time the mechanism of astrocytic scar formation involving astrocyte–SynCAM3 interactions in a mouse SCI model and shows that SynCAM3 deletion attenuated glial scar formation and restored functional outcomes after SCI.

Some limitations of this study need to be improved. First, there is not enough experimental evidence to conclusively demonstrate the mechanism in this study. In a follow-up study, we intend to solidify the mechanism elucidation of ECM repositioning over time after SCI through additional experiments such as immunofluorescence, qRT-PCR, and western blot. In addition, there is no mRNA quantification of more suitable genes (*Iba-1*, *CD45)* for microglia activation and neuronal protection-related genes (M2 macrophage; *CD163*, and *CD206*, A2 astrocyte; *S100A10*) over time. Furthermore, we did not perform hematoxylin and eosin staining, but only performed luxol fast blue staining. However, the results of this study may still provide insight into the novel therapeutic strategy for SCI using selective SynCAM3 deletion to lead to the inhibiting of glial scar formation.

## 4. Materials and Methods

### 4.1. Subjects and Surgical Procedures

All animal surgical procedures were performed under the approval of the Institutional Animal Care and Use Committee (IACUC) of CHA University (IACUC200066). For this study, female 12-week-old C57BL/6 mice (20–25 g, Koatec Inc, Pyeongtaek, Korea, *n* = 50) were purchased. The mice were fully cared for under conditions of 55–65% humidity and temperature 24 ± 3 °C. The mice had free access to food and regular tap water. Mice heterozygous (cord) for SynCAM3 deficiency (C57BL/6N-Cadm3^tm1^) were purchased (*n* = 50) from the Korea Research Institute of Bioscience and Biotechnology (Ochang, Korea) and intercrossed to generate SynCAM3 KO mice. WT (C57BL/6N) mice served as controls (*n* = 50), Animals were deeply anesthetized with a mixture of Zoletil^®^ (50 mg/kg, Virbac Laboratories, Carros, France) and Rompun^®^ (10 mg/kg, Bayer, Seoul, Korea) through intraperitoneal injection. To induce a static weight compression injury, a laminectomy was made at the ninth thoracic vertebral level (T9) and a stainless-steel 20 g of impactor was loaded to the T10 spinal cord for 30 s after laminectomy. Following a compression lesion, animals were placed on a heating pad maintaining temperature until they recovered and then 0.5 mL of 0.9% sterile saline was injected subcutaneously. Cefazolin (Chong Kun Dang Pharmaceutical Co., Ltd., Asan, Korea) was injected to prevent infection and Ketoprofen (Samchondang Pharmaceutical Co., Ltd., Seoul, Korea) was injected to reduce pain through muscle injection after operation. The bladder was squeezed manually twice a day until the bladder reflex recovered.

### 4.2. qRT-PCR

Total RNA was isolated from that obtained from the injured spinal cord (2-mm-long tissue samples) using an RNeasy Micro kit (Qiagen, Hilden, Germany). For cDNA synthesis, a reverse transcription reaction was performed using a Prime Script first-strand cDNA Synthesis kit (Takara Bio, Kusatsu, Japan). qRT-PCR was performed using primers that we’re interested in experiment and the SYBR Green Master Mix (Takara Bio, Kusatsu, Japan). The primer sequence is given in Table 1. The target mRNA level was normalized to the level of the GAPDH and compared with the control. Data were analyzed using the ΔΔCT method.

### 4.3. Western Blot Analysis

Spinal cord tissues were collected at DPI-1 and DPI-28 and washed with ice-cold phosphate-buffered saline, placed at 4 °C, homogenized in 180 lysis buffer (PRO-PREP™, iNtRON Biotechnology, Seongnam, Korea), and centrifuged at 14,000 rpm at 4 °C for 15 min. The supernatant was collected to determine protein concentration using a Bio-Rad DC Protein Assay (Bio-Rad, Hercules, CA, USA). Protein concentration was determined by a VersaMax™ microplate reader (Molecular Devices, San Jose, CA, USA). Equal amounts of protein (40 μg) were separated electrophoretically by 10% sodium dodecyl sulfate-polyacrylamide gel electrophoresis, and the resolved proteins were transferred to polyvinylidene fluoride membranes (#162-0177, Bio-Rad, Hercules, CA, USA). The membranes were then incubated for 1 h with 5% non-fat skim milk prepared in TBS buffer to block nonspecific binding. The membranes were then incubated overnight in the cold room with primary antibodies after 1-h incubation at room temperature with corresponding secondary antibodies. The blots were visualized with enhanced chemiluminescence (ECLTM, GE Healthcare, Chicago, IL, USA), using the LAS 4000 biomolecular imager (GE Healthcare, Chicago, IL, USA). The immunoblotting was quantified using ImageJ software (Fiji).

### 4.4. Behavioral Analyses

Motor function was evaluated using the locomotor open-field rating scale on the BMS. The scoring was from 0 points to 9 points. All tests were performed in a double-blinded fashion.

### 4.5. Histopathological Examination

Twenty-eight days after SCI surgery, animals were anesthetized and perfused with 0.9% saline and fixed with 4% paraformaldehyde. After removal of the spinal cord from the body, the tissue was placed in 30% sucrose until the tissue for overnight or until tissue sinks. The tissue was then embedded in an optimal cutting temperature (OCT) compound. The frozen sections were cut in the sagittal plane at 16 μm or the axial plane at 20 μm. The sections were subsequently stained with primary antibodies in at 4 °C overnight and were incubated with Alexa Fluor–conjugated secondary antibodies (1:200; Invitrogen, Waltham, MA, USA) for 1 h at room temperature and next day. The following primary antibodies were used: CSPGs (mouse specific, 1:200, Abcam, Cambridge, UK, ab11570), Collagen type 1 (mouse specific, 1:200, Abcam, Cambridge, UK), GFAP (1:200, mouse/rat specific, 1:200, Abcam, Cambridge, UK, ab10062), Cadm3 (mouse specific, 1:200, SYSY, Göttingen, Germany, 243 303). Sections were mounted in Dako fluorescence mounting medium (Dako, S3023, Glostrup, Denmark) and examined using a Zeiss Axioscan 7 (Carl Zeiss, Jena, Germany, or Leica, Wetzlar, Germany). Image quantification for immunofluorescence signals was performed using Zen 3.0. An equal area (nm^2^) was selected, channel intensities were measured, and the fluorescence intensity mean value (IMV) was obtained. The fluorescence IMV was plotted as mean ± SEM in Origin8.5.

### 4.6. LFB Staining

LFB staining was performed on the spinal cord tissue to evaluate the demyelination using a Luxol Fast Blue Stain Kit (Abcam, Cambridge, UK, #ab150675) at 28 DPI. The lesion sections of the spinal cord were deparaffinized in Xylene (Samchun Chemical, Pyungtaek, Korea) and dehydrated in ethanol solution that was graded and incubated in LFB solution at room temperature overnight. The samples were differentiated in a lithium carbonate solution. Scanning was conducted using an Olympus C-mount camera adapter (U-TVO.63XC, Tokyo, Japan).

### 4.7. RNA Isolation

Total RNA was isolated using TRIzol reagent (Invitrogen, Waltham, MA, USA). RNA quality was evaluated using an Agilent 2100 bioanalyzer (Agilent Technologies, Amstelveen, Netherlands), and RNA quantification was performed using an ND-2000 Spectrophotometer (Thermo Inc., Waltham, MA, USA).

### 4.8. Library Preparation and Sequencing

The library was prepared on a total RNA using the NEBNext Ultra II Directive RNA-Seq Kit (New England BioLabs, Inc., Ipswich, MA, UK). The isolation of mRNAs was performed with a Poly(A) RNA Selection Kit (LEXOGEN, Inc., Vienna, Austria). The isolated mRNA was used for cDNA synthesis and shearing to the manufacturer’s instructions. Indexing was performed using an Illumina index 1–12. The enrichment step was performed using PCR. The library was verified using the Agilent 2100 bioanalyzer (DNA High Sensitivity Kit) to evaluate the average fragment size. The library quantification kit and Step One Real-Time PCR System (Life Technologies, Inc., Carlsbad, CA, USA) were used for quantification. High throughput sequencing was carried out with paired-end 100 sequencing using a Nova Seq 6000 (Illumina, San Diego, CA, USA).

### 4.9. Visium Experiment Methods

Frozen samples were embedded in OCT compound (25608-930, VWR, Radnor, PA, USA) and sectioned at −20 °C with a cryotome (Thermo Scientific, Waltham, MA, USA). Tissue sections were placed on chilled Visium Tissue Optimization Slides (1000193, 10X Genomics, Pleasanton, CA, USA) and Visium Spatial Gene Expression Slides (1000184, 10X Genomics, Pleasanton, CA, USA), and were attached to the slide by warming on a heating block. We fixed the sections onto the slide in chilled methanol for staining under the Visium User Guide (10X Genomics, Pleasanton, CA, USA). For gene expression analysis, cDNA libraries were prepared according to the Visium Spatial Gene Expression User Guide and were sequenced on a NovaSeq 6000 System S1 200 (Illumina, San Diego, CA, USA) at a sequencing depth of up to 250M read-pairs per sample.

### 4.10. Library Kit

- Visium Spatial Expression Slide & Reagent Kit, 16rxn PN-1000184
- (-Spatial Gene Expression Slide Kit, -Spatial Gene Expression Reagent Kit, -Library Construction Kit)
- Visium Gateway Package, 2 rxns PN-1000316
- Visium Gateway Slide, 2 rxns PN-1000317
- Visium Accessory Kit, PN-1000194
- Dual Index Kit TT Seat A, 96 rxns PN-1000215

### 4.11. Visium BI Method

Raw FASTQ files and histology images were processed by sample with Space Ranger v1.1.0 software, which uses STAR v.2.5.1b (Dobin et al., 2013) for genome alignment, against the Cell Ranger hg38 reference genome “refdata-gex-GRCh38-2020-A”. Available online: https://cf.10xgenomics.com/supp/cell-exp/refdata-gex-GRCh38-2020-A.tar.gz (accessed on 11 April 2022) [51]. The alignment and count processes were performed by the ‘spaceranger count’ command, specifying the input of FASTQ files, reference, section image, and Visium slide information. The pipeline detects the tissue area by aligning the image to the printed fiducial spot pattern of a Visium slide and recognizing stained spots from the image. Spatially located transcriptomic data were analyzed by Seurat v3.1.3 for quality measurement and clustering. The quality of each spot was evaluated by the UMI count, the number of expressed genes, and the percentage of mitochondrial gene expression. The gene expression matrix was then normalized and scaled for dimension reduction and clustering analysis. To access the quantified expression data and images interactively, the coordinates of dimension reduction and clustering results were transformed in the Loupe Browser, available online: https://support.10xgenomics.com/single-cell-geneexpres-sion/software/downloads/latest#loupetab (accessed on 11 April 2022). Visualized information was used to distinguish differentially expressed genes between clusters or between anatomically distinct locations.

### 4.12. Data Analysis

Quality control of the raw sequencing data was performed using FastQC (Simon, 2010). Adapter and low-quality reads (<Q20) were removed using FASTX_Trimmer (Hannon Lab, 2014) and BBMap (Bushnell, 2014). The trimmed reads were then mapped to the reference genome using TopHat (Cole Trapnell et al., 2009). Gene expression levels were estimated using FPKM (Fragments Per kb per Million reads) values by Cufflinks (Roberts et al., 2011). The FPKM values were normalized based on the Quantile normalization method using EdgeR within R (R development Core Team, 2016). Data mining and graphic visualization were performed using ExDEGA (Ebiogen Inc., Seoul, Korea).

### 4.13. Statistical Analysis

For statistical analysis, one-way analysis of variance (more than three samples) or the Student’s *t*-test (two samples) was used. Data are presented as means ± standard error of the mean (SEM). A *p-*value < 0.05 was considered to indicate statistical significance.

## Figures and Tables

**Table 1:** Primer sequence for qRT-PCR.

Primers	Directions	Sequence
*Acan*	Forward	5′-TTTGATTTCCCACCGTGCCTTTCC-3′
	Reverse	5′-TTCCTGGTCCTGTCTTCTTTCAGC-3′
*Axin2*	Forward	5′-GAGAAGTTTGGATTGCTGTCCACG-3′
	Reverse	5′-ACCCATTTCTGCATGTGTCGATGG-3′
*Bcan*	Forward	5′-AATTCTGCTGAAGGCTCAATGCCC-3′
	Reverse	5′-CGGAAGTGACAGAATGGAAGATCC-3′
*Cdh2*	Forward	5′-TACGCAGCTGGTTGCAGATAAAGG-3′
	Reverse	5′-TCTGCACTCCTCCATAGTCTATGC-3′
*Chst11*	Forward	5′-GTTCTGGTGAAGTCCACAAACTGC-3′
	Reverse	5′-TGTCACCATGGGATTCTACACACG-3′
*Cnpase*	Forward	5′-AAATGGCAGACCAGTATCAGTACC-3′
	Reverse	5′-GTCTCAGAACTCTTTTTGGTCAGG-3′
*Col1a1*	Forward	5′-CATGGAGACAGGTCAGACCTGTGT-3′
	Reverse	5′-GGACATTAGGCGCAGGAAGGTCAG-3′
*Col1a2*	Forward	5′-ATCCAAC TAAGTCTCCT CCCTTGG-3′
	Reverse	5′-GGCTTCTGACTATCTTCCACAGAG-3′
*Csgalnact1*	Forward	5′-CTTGAGACAGTCTTGTCACAGAGC-3′
	Reverse	5′-CAGTCCTTAGATCAGATCTCCAGG-3′
*Ctnnb1*	Forward	5′-GGGTGAATACTTTACTCTGCCTGC-3′
	Reverse	5′-GTATAACGCTGCAAAAGCTGTGGC-3′
*Gapdh*	Forward	5′-GACTTCAACAGCAACTCCCACTCT-3′
	Reverse	5′-GGTTTCTTACTCCTTGGAGGCCAT-3′
*Gfap*	Forward	5′-TGTACTAACAGAGCGAGCCTATGC-3′
	Reverse	5′-GGGACTTGCTGCCTTTAACATTGG-3′
*lba1*	Forward	5′-CAAAGAACACAAGAGGCCAACTGG-3′
	Reverse	5′-TTCCATGCTGCTGTCATCAGAAGC-3′
*Mmp13*	Forward	5′-GAGAGCTTAGTTCTGTGAACGAGC-3′
	Reverse	5′-AAAGCAGATGGACCCCATGTTTGC-3′
*Mmp2*	Forward	5′-CTATCATCTTCATCGCTGCACACC-3′
	Reverse	5′-GTACAGTCAGCACCTTTCTTTGGG-3′
*Mmp9*	Forward	5′-AAGGTATTCAGTTGCCCCTACTGG-3′
	Reverse	5′-ACACGGAGAATCTCTGAGCAATCC-3′
*Nefh*	Forward	5′-TAGCAAGAGAAGATAACCCTGAGC-3′
	Reverse	5′-TCATCTGTCAGTTGGACATACAGG-3′
*Nes*	Forward	5′-GTCAGCTGAGCCTATAGTTCAACG-3′
	Reverse	5′-AGAGTCACTCATCATTGCTGCTCC-3′
*Pcan*	Forward	5′-TAATGGTGCAGCTTTGCCTGATGG-3′
	Reverse	5′-CCTGACAGTAACTCATTCTGCTGC-3′
*Pdgfa*	Forward	5′-AGACAGATGTGAGGTGAGATGAGC-3′
	Reverse	5′-ACGGAGGAGAACAAAGACCGCACG-3′
*Pdgfb*	Forward	5′-TACCTCCACTCTGTGTCTTCTTCC-3′
	Reverse	5′-CATCCCATTACAACCTTGCTCACC-3′
*Plaur*	Forward	5′-TCTGGATCTTCAGAGCTTTCCACC-3′
	Reverse	5′-CTTACGGTATAACTCCGGTTTCCC-3′
*Slit2*	Forward	5′-CGTCTCTAGAAGCTTCTAGCTTCG-3′
	Reverse	5′-TGTAGGGGGAGCTTTAGTACAAGC-3′
*Sox9*	Forward	5′-GAAGGTAACGATTGCTGGGATTCC-3′
	Reverse	5′-CGTCCTCCATGTTAACTCTGAAGG-3′
*Tgfb1*	Forward	5′-GTGACAGCAAAGATAACAAACTCC-3′
	Reverse	5′-GAGCTGAAGCAATAGTTGGTATCC-3′
*Tgfb2*	Forward	5′-TCTGAGATTACAGCAACAACAACC-3′
	Reverse	5′-CAATACGTACAACTCCACTGAACG-3′
*Vim*	Forward	5′-TGCTAACTACCAGGACACTATTGG-3′
	Reverse	5′-AGGTTAGTTTCTCTCAGGTTCAGG-3′
*Xylt1*	Forward	5′-CAGTGAAGATTCTCCATCACTGGG-3′
	Reverse	5′-TCTGGAAACTCTGCTCCATGTAGG-3′

*Acan*, aggrecan; *Axin2*, axis inhibition protein 2; *Bcan*, brevican; *Cdh2*, cadherin 2; *Chst11*, carbohydrate sulfotransferase 11; *Cnpase*, 2′,3′-cyclic nucleotide 3′ phosphodiesterase; *Col1a1*, collagen, type I, alpha 1; *Col1a2*, collagen, type I, alpha 2; *Csgalnact1*, chondroitin sulfate N-acetylgalactosaminyltransferase 1; *Ctnnb1*, catenin beta 1; *Gapdh*, glyceraldehyde 3-phosphate dehydrogenase; *Gfap*, glial fibrillary acidic protein; *lba1*, ionized calcium-binding adaptor molecule 1, *Mmp13*, matrix metallopeptidase 13, *Mmp2*, matrix metallopeptidase 2; *Mmp9*, matrix metallopeptidase 9; *Nefh*, neurofilament heavy chain; *Nes*, nestin; *Pcan*, phosphacan; *Pdgfa*, platelet derived growth factor subunit A; *Pdgfb*, platelet derived growth factor subunit B; *Plaur*, plasminogen activator, urokinase receptor; *Slit2*, slit guidance ligand 2; *Sox9*, SRY-Box transcription factor 9; *Tgfb1*, transforming growth factor beta 1; *Tgfb2*, transforming growth factor beta 2; *Vim*, vimentin; *Xylt1*, xylosyltransferase 1.

## Data Availability

Data available in a publicly accessible repository.
